# The Relationship between Masculinity and Internalized Homophobia amongst Australian Gay Men

**DOI:** 10.3390/ijerph17155475

**Published:** 2020-07-29

**Authors:** Jack Thepsourinthone, Tinashe Dune, Pranee Liamputtong, Amit Arora

**Affiliations:** 1 School of Health Sciences, Western Sydney University, Sydney 2751, Australia; t.dune@westernsydney.edu.au (T.D.); p.liamputtong@westernsydney.edu.au (P.L.); a.arora@westernsydney.edu.au (A.A.); 2 Translational Health Research Institute, Western Sydney University, Sydney 2751, Australia; 3 Oral Health Services, Sydney Local Health District, Sydney 2010, Australia; 4 Sydney Medical School, The University of Sydney, Sydney 2050, Australia

**Keywords:** internalized homophobia, homonegativity, masculinity, LGBT, gender norms

## Abstract

Due to the heterosexist ideals associated with gender norms, gay men often experience negative attitudes towards their own sexuality—internalized homophobia. As a result, gay men often feel compelled to compensate for their perceived lack of masculinity. The study aimed to investigate the relationship and predictive power of masculinity on gay men’s experiences of internalized homophobia. A sample of 489 self-identified Australian gay men 18–72 years old participated in an online survey on masculinity and homosexuality. Descriptive statistics, bivariate correlations, and sequential multiple regressions were used to test the study’s aims. Sequential multiple regressions revealed that conformity to masculine norms and threats to masculinity contingency were stronger predictors of internalized homophobia over and above demographic and other factors. Given the already known psychological risks associated with social isolation, internalized homophobia, and the poor mental health outcomes associated with sexual minority groups, it is suggested that gay men who are experiencing high degrees of internalized homophobia should not be distancing themselves from other gay men but, conversely, seek a strong relationship with the LGBTI community.

## 1. Introduction

Like any individual, the socio-ecological environment of a gay man includes a complex network ranging from the macrosystem—including broader social structures and ideologies—to the microsystem—including their family and close social networks which progressively shapes (and is shaped by) the individual [1,2,3]. Within the gay male community, heteronormative ideals play a prominent role—the rewarding of traditionally masculine behavior and stigmatization of effeminate behavior [4]. As a consequence, sexual minority individuals often experience negative attitudes towards their own sexuality—internalized homonegativity [5].

Gay men often experience a higher degree of negative attitudes, abuse, and extreme states of mind in response to internalized homophobia (e.g., suicide and homicide) [6,7,8,9] as well as greater violence and discrimination based on gender norm violation as compared to lesbian, bisexual, and genderqueer women who tend to benefit from such deviations [10]. Furthermore, numerous studies have made connections between internalized homophobia and depression, poor wellbeing, sexual discrimination, shame, body dissatisfaction, eating disorders, and suicidal ideation [11,12,13,14,15]. Similarly, unlike cisgender men, transgender men experience a reduction in mental health issues and more positive wellbeing outcomes in relation to conformity to masculine norms [16]. Berg et al. [12] identified that, while most literature on internalized homophobia dating 1989-2012 examines the relationship between internalized homophobia and other factors, there exists little to no empirical research on the relationship between masculine norms and internalized homophobia. Furthermore, only a maximum of eight papers were identified to originate from Australia [12]. Since this, Provence et al. [17] identified key issues around heterophobia, internalized homophobia, and the process of coming out when examining relational barriers between straight and gay men and establishing links to gendered norms. Given the negative mental health outcomes associated with internalized homophobia and the sparsity of research explicitly examining the role and relationship of masculine norms on internalized homophobia, further investigation to identify the socio-ecological underpinnings of internalized homophobia is warranted in order to better predict the salience and impact of internalized homophobia among Australian gay men across the ecology of their environment.

### 1.1. Masculinity

Manhood and masculinity, unlike womanhood and femininity, are impermanent and tenuously maintained [18]. In traditional Western and heteronormative contexts, women are expected to be passive, sentimental, and emotive whilst men are expected to be aggressive, stoic, and brave [19]. Heteronormative ideals which polarize masculinity and femininity are argued to motivate men’s maladaptive behaviors, negative mental health outcomes, feelings of inferiority, overcompensation, and contribute to men’s fear of femininity and concerns around anti-effeminacy, success, power, and competition, restrictive emotionality, and risk-taking [4,18,20,21]. However, there exists the common perception of effeminate men (regardless of actual sexual identity) as being homosexual—that is, homosexuality is equivalent to femininity [17,22]. This arguably runs the risk of also polarizing perceptions of masculinity and homosexuality.

### 1.2. Homophobia

Homophobia (or homonegativity) is conceptualized as the fear or hatred of homosexuality and the fear of being a homosexual [23,24]. This definition provides a more holistic perception of homophobia as it considers the experiences of both straight and gay individuals (i.e., internalized homophobia). However, when considering notions of homosexuality and femininity being synonymous, the definition of homophobia can, arguably, be revised to being “the fear of femininity and the fear of being effeminate”.

Falomir-Pichastor and Mugny [25] aimed to explain homophobia through social identity theory and the relationship between in- and out-groups. It was argued that gay men pose a threat to hegemonic masculinity and, therefore, straight men are motivated to maintain a distinct gender identity. This was further highlighted in Martínez, Vázquez, and Falomir-Pichastor’s [26] study whereby links to anti-effeminacy were made. Similarly, when having their masculinity threatened, masculine-identifying men tend to rate in-group members (masculine men) as more likeable than outgroup members (effeminate men) and are less likely to interact with out-group members [27,28]. When considering the previous definition of homophobia in conjunction, it can be argued that similar effects may be present within gay men—that is, can internalized homophobia be explained through gay men’s motivation to distinguish themselves from other (stereotypical) gay men through the conformity of masculinity and anti-effeminacy ideals?

Masculine behavior among gay men is commonly referred to as “straight-acting” and is argued to be an emulation of heteronormative masculinity—and, arguably, heterosexuality [29,30]. Similar to heteronormative masculinity, straight-acting masculinity is inclusive of anti-effeminacy ideals and homophobia [30]. For instance, prejudice between straight-acting and effeminate gay men is normalized and even glorified within the gay community and is often perpetrated by others who were previously discriminated against for gender non-conformity [31,32]. This heteronormative approach to masculinity—harassment due to gender non-conformity—is shown to predict later adult life body shame and anxiety among gay men [33,34]. Clarkson [30] argued that the anti-effeminacy ideals perpetuated through hegemonic masculinities which favor heteronormative expressions of gender are jeopardizing the very diversity that the Lesbian, Gay, Bisexual, Transgender, and/or Intersex (LGBTI) community is known for.

**Figure 1:**
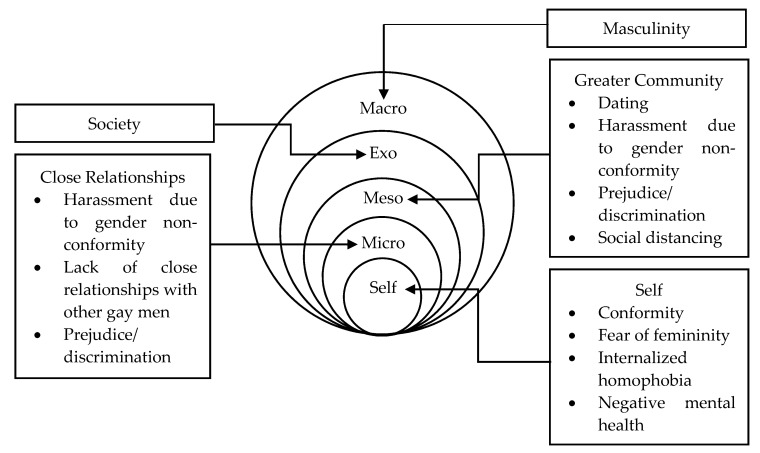
Socio-ecological map of a gay man.

Beginning from the macro level of a gay man’s social ecology, hegemonic masculinity permeates the psychology of how they perceive themselves, others, and the world—including the fear of male effeminacy and the synonymous perceptions of homosexuality and femininity [17,24]. Moreover, the interactions between the individual and their micro-, meso-, and exo-systems arguably contribute further to the issues presented [4,26,32]. Based on the literature discussed above, Figure 1 depicts a typical ecological environment for a gay man and highlights various areas hegemonic masculinity pervades and exerts an influence. It can, therefore, be argued that the role of hegemonic masculinity on a gay man’s life cannot be examined in isolation but, rather, as a complete system of variables—each a contributing and subsequent factor in the manufacturing of internalized homophobia.

### 1.3. Present Study

Despite common understandings that masculinity relates to (and possibly produces) negative attitudes toward homosexuality, there exists limited research explicitly examining masculinity and internalized homophobia [15,35,36]. The present study, therefore, aims to examine the relationship between masculinity and internalized homophobia within a sample of Australian gay men through Bronfenbrenner’s [1] socio-ecological perspective. It aims to explore:

1. The degree to which gay men conform and/or value masculine norms and whether it relates to internalized homophobia, and
2. When controlling for demographic and other factors, does the degree of conformity/valuation of masculine norms predict internalized homophobia.

## 2. Materials and Methods

### 2.1. Procedure

The Human Research Ethics Committee of Western Sydney University (Australia) approved this study (approval number: H12044) prior to its implementation. An online survey (hosted by Qualtrics) was utilized and made available through a hyperlinked text within the study’s advertisement. Individuals participated voluntarily and were informed that they may withdraw from the study at any given time. The details of the study (e.g., the Participant Information Sheet) were provided on the first page of the survey. The Participant Information Sheet provided details about the study, participant requirements, duration, use of data, and contact details of the supervisor if required under any circumstances. Once the participants read the Participant Information Sheet, they were informed that clicking on the “continue” button below the Participant Information Sheet constituted their consent to participate in the survey.

### 2.2. Participants

For this study, participants were recruited via advertisements through LGBTI networks (e.g., LGBTI Alliance of Australia, Queensland Aids Council; 4.3%), social media (e.g., Facebook, Twitter, Instagram; 8.4%), dating applications (e.g., Grindr; 84.9%), flyers placed across Western Sydney University campuses (1%), and word of mouth (1.4%). A sample of 489 self-identified Australian gay men over the age of 18 (M = 36, SD = 12.20) participated in an online survey on masculinity and homosexuality. Those identifying as transgender, bisexual, etc. were excluded from the study as it aims to assess and compare results from a single group identity (gay men). Gay men are argued to be most adversely affected by heteronormative constructions of masculinity [4,15] and femininity, are more prone to resultant health and wellbeing complications [18,37], and are more likely to experience violence and discrimination based on gender norm deviation than lesbian, bisexual, and genderqueer women, as well as transgender men [10].

### 2.3. Measures

#### 2.3.1. Demographics

A demographic questionnaire was utilized to ascertain participant’s background information—age, gender, ethnicity, post code, religion, and sexual orientation. Table 1 depicts the sample’s demographic.

#### 2.3.2. Internalized Homophobia

To measure internalized homophobia, the Internalized Homonegativity Inventory (IHNI) [5] was utilized. It consists of 23 items and measures the individuals’ Personal Homonegativity, Gay Affirmation, and Morality of Homosexuality using a 6-point Likert scale ranging from 1 (Strongly Disagree) to 6 (Strongly Agree). Items involve identifying the degree of one’s agreeability to a statement (e.g., “I feel ashamed of my homosexuality”). The IHNI is a reliable and valid measure of internalized homophobia in gay men and was developed in order to address limitations of content validity within previous scales measuring internalized homophobia [5]. The scale showed good internal consistency (Cronbach’s *α* = 0.93).

#### 2.3.3. Conformity to Masculine Norms

The Conformity to Masculine Norms Inventory-46 (CMNI-46) [38] was used to measure participants’ current conformity to masculine norms. The scale includes 46 items and measures individuals’ conformity to masculine norms along nine subscales: Emotional Control, Winning, Playboy, Violence, Self-reliance, Risk-taking, Power over Women, Primacy of Work, and Heterosexual Self-presentation. Items involve identifying the degree of one’s agreeability to a statement (e.g., “It bothers me when I have to ask for help”) using a 4-point Likert scale ranging from 1 (Strongly Disagree) to 4 (Strongly Agree). The CMNI-46 is a reliable and valid measure of males’ conformity to masculine norms and has omitted several items possessing poor construct specificity from the original Conformity to Masculine Norms Inventory [38,39]. Although Hammer, Heath, and Vogel [40] advised against the use of the scale as unidimensional, the present study’s scope examines masculine norms as a whole as opposed to specific dimensions. The total scale has also been utilized by various studies assessing men’s masculinity [41] but, most particularly, gay men’s masculinity as well [20,42]. The scale showed good internal consistency (Cronbach’s *α* = 0.81).

#### 2.3.4. Masculinity Contingency

The Masculinity Contingency Scale (MCS) [43] is a scale assessing a man’s self-worth in relation to his sense of masculinity (e.g., “I can’t respect myself if I don’t behave like a ‘real man’”). The MCS consists of 10 items and measures threats to masculinity (MCS-Threat) and boosts to masculinity (MCS-Boost) using a 7-point Likert scale ranging from 1 (Strongly Disagree) to 7 (Strongly Agree). Both the MCS-Threat (Cronbach’s *α* = 0.94) and MCS-Boost (Cronbach’s *α* = 0.95) demonstrated excellent internal consistency.

#### 2.3.5. Childhood Gender Non-Conformity

To measure participants’ past conformity to masculine norms, the seven item Childhood Gender Non-Conformity Scale (CGNcS) [29] was used. It assesses childhood harassment due to gender non-conformity. Individuals are asked to rate their agreement to a statement along a 7-point Likert scale ranging from 1 (Strongly Disagree) to 7 (Strongly Agree). An item may state “As a child I was called a ‘sissy’ by my peers”. The scale showed good internal consistency (Cronbach’s *α* = 0.84).

#### 2.3.6. Perceived Distance

An adapted single item scale from [25] will be used to assess individuals’ perceived distance from (other) gay men (i.e., it is likely that someone would think I am a homosexual). In other words, it measures the degree an individual perceives themselves to conform/deviate from the heteronormative perception of homosexuality. The scale utilizes a 7-point Likert scale ranging from 1 (Very Unlikely) to 7 (Very Likely).

#### 2.3.7. Perceived Similarity

Similarly, a seven-item scale was used to assess individuals’ perceived similarity with (other) gay men [44]. Using a 7-point Likert scale ranging from 1 (Absolutely Not) to 7 (Absolutely), individuals are asked “To what extent do you think you are similar to gay men with regard to each of the following aspects?”—Emotions, Needs, Wishes, Intimate Relationships, Friendships, Professional Relationships, and General Similarity. The scale showed good internal consistency (Cronbach’s *α* = 0.86).

### 2.4. Statistical Analyses

Statistical analyses were conducted using Statistical Package for Social Science (SPSS; IBM, Sydney, Australia) version 22 and, following data collection, participant data was screened for missing data (participants who withdrew from the study were excluded), as well as univariate and multivariate outliers. Using the ± 3.29 *z* score criteria, 62 univariate outliers were identified and deleted from the sample. Additionally, with alpha set at 0.001, two multivariate outliers with Mahalanobis distance scores above 43.82 were identified and deleted. Once the data was screened, 489 participant datasets remained.

Descriptive statistical analyses were used to examine the degree gay men conform to measures of masculinity, internalized homophobia, and other variables. Bivariate correlations were used to examine relationships between demographic, internalized homophobia, measures of masculinity, and interpersonal factors. Furthermore, sequential multiple regression analyses were conducted using three models beginning with demographic variables and inserting interpersonal variables and measures of masculinity, respectively, at each step.

## 3. Results

The study’s sample included 489 self-identified Australian gay men 18–72 years of age (M = 36, SD = 12.20). In viewing the sample means alone, it can be seen that participants tend to conform neither extremely highly nor extremely lowly to masculine norms (M = 95.82, SD = 11.46). Additionally, participants tended not to value masculinity very highly where MCS-Threat (M = 11.22, SD = 6.40) and MCS-Boost (M = 18.92, SD = 8.04). Table 2 displays the participant mean scores in all measured variables.

Pearson product-moment correlations were performed between the variables age, education, Internalized Homophobia, Conformity to Masculine Norms, Masculinity Contingency-Threat, Masculinity Contingency-Boost, Childhood Gender Non-Conformity, Perceived Distance, and Perceived Similarity using an alpha level of 0.05. As the sample was large (*n* = 489), assumptions of homogeneity and variance were satisfactory. Table 3 depicts correlations between all test variables.

A sequential multiple regression was conducted in order to determine whether the variables (age, education, Conformity to Masculine Norms, Masculinity Contingency-Threat, Masculinity Contingency-Boost, Childhood Gender Non-Conformity, Perceived Distance, and Perceived Similarity) could predict Internalized Homophobia. With an *n* of 489, assumptions of homogeneity were satisfactory. Results of the multiple regression are displayed in Table 4.

Model 1, F (2,486) = 7.70, *p* < 0.001, shows the association between demographic information and internalized homophobia whereby age is a significant predictor (*p* < 0.001). Model 2, F (4,484) = 13.43, *p* < 0.001, shows the association when interpersonal variables are added to the model. Age (*p* < 0.001) continues to act as a significant predictor while Perceived Distance (*p* < 0.05) and Perceived Similarity (*p* < 0.001) are also significant predictors. Additionally, Model 3, F (8,480) = 22.44, *p* < 0.001, shows the association when various measures of gender norm conformity are included in the model. Age (*p* < 0.01) and Perceived Similarity (*p* < 0.01) remain a significant predictor of internalized homophobia while Perceived Distance is no longer significant. Additionally, Conformity to Masculine Norms (*p* < 0.001) and Masculinity Contingency-Threat (*p* < 0.001) were significant predictors. The results demonstrate that age, perceived similarity, Conformity to Masculine Norms, and Masculinity Contingency in relation to threats to masculinity were sufficient to predict levels of Internalized Homophobia over and above other demographic variables, Perceived Distance to gay men, and other measures of gender norm conformity.

## 4. Discussion

In order to better understand the socio-ecological factors contributing to the development of internalized homophobia, the present study sought to examine the degree gay men conform to and/or value masculine norms. Beginning with the individual, descriptive statistics suggest that Australian gay men’s conformity to masculine norms tends to lean towards neither extremes. Additionally, Australian gay men tend not to value masculinity very highly, as suggested by their sense of self-worth in relation to threats/boosts to masculinity.

To further understand the salience of influencers from an individual’s macrosystem on the individual, the study aimed to explore the degree to which gay men’s conformity and/or valuation of hegemonic masculinity predicted the degree of internalized homophobia experienced. The results supported that only the measures of Conformity to Masculine Norms and MCS-Threat remained as significant predictors of internalized homophobia. This suggests that individuals’ current conformity to masculine norms are stronger predictors of individuals’ experiences of internalized homophobia as compared to past conformity (e.g., childhood gender non-conformity). This is contrary to other findings which suggest that childhood harassment due to gender non-conformity would predict negative later life health outcomes (e.g., body shame, anxiety, gender-related strain) [32,33,34]. Additionally, the results suggest that the significance of threats to gay men’s masculinity are superior predictors of internalized homophobia as compared to the significance of boosts to their masculinity.

The present study’s results not only suggest that individuals who conform more to masculine norms tend to possess higher degrees of internalized homophobia than those who conform less to masculine norms but also, by knowing how strongly an individual adheres to masculine norms, one may predict the degree of internalized homophobia the said individual harbors. From these findings, it is argued that either gay men who possess stronger internalizations of homophobia utilize masculinity as a compensatory strategy or that hegemonic masculinities foster internalized homophobia—resulting in masculine-conforming men to possess stronger internalized homophobia. In other words, influences exerted within an individual’s macrosystem (masculinity) may foster internalized homophobia and/or factors within the individual’s self (internalized homophobia) influence how they present themselves and interact with others in their micro- and meso-systems. It has been argued that gay men who are overly concerned with masculine norms utilize hyper-masculinity as a compensatory strategy for their perceived sense of inferiority [4,45]. Additionally, feminine men often receive more negative attitudes, social and romantic rejection, victimization, and harassment from others, as compared to masculine men [46,47,48]. This is also evident within the Asian culture (e.g., Nepal, Thailand, Vietnam) and can be argued to be consistent across broader societal contexts [10,49,50,51]. Considering the notion that homosexuality is synonymous to femininity [17], it is no surprise that gay men experience a sense of inferiority/negativity regarding their own sexuality and aim to compensate by adopting (what they perceive to be) the opposite—masculinity.

Considering O’Neal et al.’s [24] definition of homophobia alongside Provence et al.’s [17] assertion that homosexuality is synonymous to femininity, it is argued that, as a compensatory strategy, gay men who possess higher degrees of (internalized) homophobia reduce their likelihood of being perceived as gay by conforming to what they perceive as being the antithesis of homosexuality—masculinity. This (arguably hyper-masculine) behavior can be described as straight-acting. The term is argumentative, in itself, as it is perplexing how gay men may refer to themselves as straight-acting rather than masculine. This suggests that there exists a pervasive ideology that masculinity is exclusive to heterosexuality—or in other words, masculinity is reified as the heterosexual male.

As it is maintained that male heterosexuality is perceived as being synonymous to masculinity, it is also maintained that male homosexuality is perceived as being synonymous to femininity [17]. This societal understanding of gender and sexuality, therefore, presents an oxymoron in the phrase “gay male”. By this notion, the two terms “gay” and “male” can be regarded as direct oppositions of each other. It is no wonder that gay men may experience gender discrepancy strain. In conjunction with the present study’s results, it is argued that gay men experience all three types of gender-related strain. Having traced this issue back to influences within the individual’s macrosystem, it is, therefore, argued that heteronormative understandings of gender and sexuality need to be revised in order for the strain experienced by gay men to be adequately addressed.

Additionally, perceived similarity remained a significant predictor of internalized homophobia whereby individuals who harbored higher degrees of internalized homophobia tended to perceive themselves as dissimilar to other gay men. This corroborates Falomir-Pichastor and Mugny’s [25] theory explaining homophobia through social identities—in- and out-groups. It was argued that gay men pose a threat to the masculine identity and, therefore, straight men are motivated to maintain a distinct gender identity. Similarly, masculine men tend to show prejudice and disinterest in interacting with effeminate men [27,28]. Therefore, just as straight men with higher degrees of homophobia dissociate their gender identities from gay men, it can be argued that so too do gay men with higher degrees of internalized homophobia perceive themselves as dissimilar to other gay men. Amongst a demographic considered a minority, the distancing of the self from other gay men can be considered a distressing social phenomenon.

**Figure 2:**
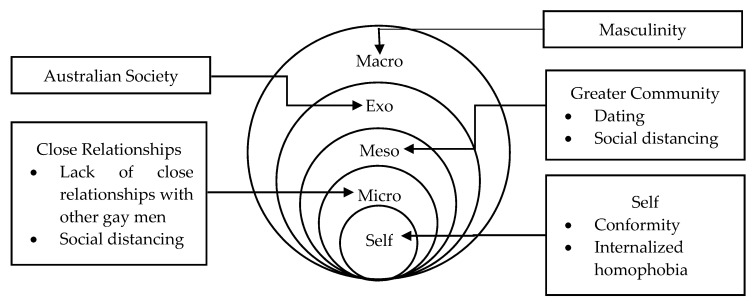
Socio-ecological map of a gay man.

Figure 2 depicts the socio-ecological system relevant to the study’s participants and highlights various areas where hegemonic masculinity pervades and exerts an influence. Although most gay men’s microsystem consists mostly of non-LGBTI friends [52,53], strong interpersonal relationships with other LGBTI individuals and having a sense of belonging with the gay community are argued to alleviate psychological distress and depression [54,55]. Similar positive effects are evident among transgender individuals [56]. Given the psychological risks associated with social isolation, internalized homophobia, and the poor mental health outcomes associated with sexual minority groups [11,12,13,14,15,16], it is suggested that, not only gay men but, LGBTI individuals who are experiencing high degrees of internalized homophobia should not be distancing themselves from other LGBTI individuals but, conversely, seek a strong relationship with them.

In addition to masculinity, analyses revealed that age predicted internalized homophobia, whereby older gay men, as compared to their younger counterparts, tend to experience lower degrees of internalized homophobia. This is consistent with other studies which found internalized homophobia and negative health and psychological outcomes to decrease with age while resiliency increases [52,57]. This, however, was not true for gay men who recently came out [57]. It is maintained that the common phrase “it gets better” holds true in regard to gay men’s experiences of internalized homophobia. It is, therefore, argued that programs and policies in support of individuals who have recently come out (e.g., gay youths) should be improved and advocated in the aim of reducing/mitigating the effects of internalized homophobia.

### Limitations

A limitation of the study may include the vagueness of the Perceived Similarity and Perceived Distance scales which do not specify whether the other individual is feminine/masculine, introverted/extroverted, or any other personality traits. Previous studies [28] provided vignettes describing the individual in question. The present study, however, assumes participants to possess similar conceptions of masculinity and the stereotyped gay man (i.e., effeminate). However, previous studies have demonstrated individuals (regardless of gender or sexuality) to possess similar conceptions of gay men [58].

Additionally, the present study’s sample only included individuals identifying as gay. Participants recruited via the dating app, Grindr, comprised 84.90% of the total sample. However, prior to eliminating data from those who do not meet the sample criteria, the study received data from participants identifying as bisexual and, surprisingly, heterosexual. It can be argued that some same-sex attracted males who identify as heterosexual may be experiencing conflicts with internalized homophobia and have not yet come to terms with their sexual identity. Therefore, it can be argued that the study’s results may only be representative of homosexual men who are comfortable with their sexual identity and, therefore, do not suffer extremely from internalized homophobia. Additionally, although the present study did not examine transgender and genderqueer men, it is recognized that gender norms operate under a different framework for transgender men (and women) compared to cisgender men (and women) whereby conformity and non-conformity may be experienced positively [16]. It is recommended that future studies include other LGBTI identities as this would allow for a more diverse range of scores. By examining other LGBTI identities, it is expected that studies can better understand the scope and impact internalized homophobia has on LGBTI individuals. Findings may come to contribute to the development of more informed strategies aimed at improving the social, psychological, and physical health outcomes of LGBTI individuals.

Additionally, the present study’s analyses could arguably provide more statistical power had the outliers and missing data not been excluded and addressed by using methods such as conducting multiple imputations [59,60]. However, this method is argued to contribute further to any biases, including self-report and social desirability bias, already present within the sample and decrease reliability [59,61]. Although bias was controlled, Bullock and Ha [62] argued that it is impossible to examine non-experimental data completely without bias. Additionally, the present study was exploratory, and it is acknowledged that results from non-experimental designs can only be interpreted as suggestive as opposed to causal [63]. Future research is recommended to examine internalized homophobia and masculinity using an experimental design.

## 5. Conclusions

Currently, there exists limited research explicitly examining masculinity and internalized homophobia [15,35,36]. The current paper is an exploration into the phenomenon of internalized homophobia and its socio-ecological underpinnings whereby discussion focused mainly on factors within the micro-, meso-, and macro-systems. Future studies, however, may wish to examine factors outside of those discussed and may wish to examine other LGBTI identities. Conceptions of heteronormative masculinity are argued to contribute negatively to non-heteronormative (LGBTI) individuals who abide by them whilst engagement with LGBTI communities is argued to support more positive outcomes for gay men and alleviate gender-related strains experienced. It is hoped that the current findings contribute to the empirical study of masculinity in the context of LGTI individuals and the development of policies and support services, notably LGBTI youth. Future studies are urged to expand upon the knowledge and understanding acquired from the present study, particularly in relation to internalized homophobia and interpersonal relations.

## Figures and Tables

**Table 1:** Demographic information for the study sample (*n* = 489).

Demographic Information	(%)
***Education***	
No Formal Education	0.20
Secondary	4.50
Higher School Certificate	6.30
Diploma/Certificate	17.80
Bachelor	39.90
Post-graduate	31.30
***Religious affiliation***	
Buddhism	4.30
Christianity	23.90
Hinduism	0.80
Islam	0.20
Judaism	0.80
No Religion	66.40
Other	3.70

**Table 2:** Descriptive statistics for the study sample.

Variables	M	SD	Possible Range
IHNI	42.25	13.28	23–138
CMNI	95.82	11.46	46–184
MCS-Threat	11.22	6.40	5–35
MCS-Boost	18.92	8.04	5–35
Childhood Gender Non-Conformity	26.76	9.90	7–49
Perceived Distance	4.58	1.76	1–7
Perceived Similarity	33.97	7.77	7–49

Note. IHNI = Internalized Homophobia; CMNI = Conformity to Masculine Norms; MCS-Threat = Masculine Contingency-Threat; MCS-Boost = Masculine Contingency-Boost.

**Table 3:** Bivariate correlations between demographic and various factors.

Variables	Variables								
	Age	Education	IHNI	CMNI	MCS-T	MCS-B	CGNcS	Distance	Similarity
Age	—								
Education	0.06	—							
IHNI	−0.16 **	−0.08	—						
CMNI	−0.14 **	−0.04	0.39 **	—					
MCS-Threat	−0.02	−0.05	0.42 **	0.40 **	—				
MCS-Boost	−0.12 **	0.02	0.26 **	0.29 **	0.55 **	—			
CGNcS	−0.23 **	0.04	−0.02	−0.01	−0.12 **	−0.03	—		
Distance	−0.15 **	0.06	−0.14 **	−0.09	−0.18 **	0.01	0.44 **	—	
Similarity	0.02	−0.02	−0.24 **	−0.25 **	−0.15 **	−0.07	0.13 **	0.23 **	—

** *p* < 0.01 level.

**Table 4:** Regression coefficients of the predictors on internalized homonegativity.

Variables	Model 1			Model 2			Model 3		
	*B*	SE *B*	*β*	*B*	SE *B*	*β*	*B*	SE *B*	*β*
Age	−0.017 ***	0.05	−0.16	−0.19 ***	0.05	−0.17	−0.13 **	0.05	−0.12
Education	−0.81	0.55	−0.07	−0.78	0.53	−0.06	−0.57	0.48	−0.05
Perceived Distance				−0.87 *	0.35	−0.11	−0.57	0.35	−0.07
Perceived Similarity				−0.37 ***	0.08	−0.21	−0.22 **	0.07	−0.13
CMNI							0.25 ***	0.05	0.22
MCS-Threat							0.61 ***	0.10	0.29
MCS-Boost							0.02	0.08	0.01
CGNcS							0.06	0.06	0.04

Note. *B* = unstandardized regression coefficient; SE *B* = standard error; *β* = standardized regression coefficient * *p* < 0.05; ** *p* < 0.01; *** *p* < 0.001.
